# Enteral administration of the protease inhibitor gabexate mesilate preserves vascular function in experimental trauma/hemorrhagic shock

**DOI:** 10.1038/s41598-023-36021-7

**Published:** 2023-06-22

**Authors:** Nathalia J. D. Moreira, Fernando dos Santos, Joyce B. Li, Federico Aletti, Maria Claudia C. Irigoyen, Erik B. Kistler

**Affiliations:** 1 Instituto do Coração, Hospital das ClínicasFaculdade de Medicina, Universidade de São Paulo, São Paulo, Brazil; 2 Department of Anesthesiology and Critical Care, University of California, San Diego, La Jolla, CA USA; 3 Department of Bioengineering, University of California, San Diego, La Jolla, CA USA; 4 Universidade Federal de São Paulo, São José dos Campos, Brazil; 5 Veterans Affairs San Diego Healthcare System, San Diego, CA USA

**Keywords:** Trauma, Circulation, Blood flow, Public health, Therapeutics

## Abstract

Preserving vascular function is crucial for preventing multiorgan failure and death in ischemic and low-pressure states such as trauma/hemorrhagic shock (T/HS). It has recently been reported that inhibiting circulating proteases released from the bowel to the circulation during T/HS may preserve vascular function and improve outcomes following T/HS. This study aimed to evaluate the role of the serine protease inhibitor gabexate mesilate (GM) in preserving vascular function during T/HS when given enterally. We studied the vascular reactivity of mesenteric arteries from male Wistar rats treated with enteral GM (10 mg/kg) (GM-treated, n = 6) or control (Shock-control, n = 6) following (T/HS) using pressure myography. Concentration–response curves of endothelial-dependent and endothelial-independent agonists (e.g., acetylcholine, sodium nitroprusside) ranging from 10^−10^ to 10^−5^ M were performed. In a second set of experiments, ex-vivo arteries from healthy rats were perfused with plasma from shocked animals from both groups and vascular performance was similarly measured. Arteries from the GM-treated group demonstrated a preserved concentration–response curve to the α_1_ adrenergic agonist phenylephrine compared to arteries from Shock-control animals (− logEC_50_: − 5.73 ± 0.25 vs. − 6.48 ± 0.2, Shock-control vs. GM-treated, p = 0.04). When perfused with plasma from GM-treated rats, healthy arteries exhibited an even greater constriction and sensitivity to phenylephrine (− logEC_50_: − 6.62 ± 0.21 vs. − 7.13 ± 0.21, Shock-control vs. GM-treated, p = 0.02). Enteral GM also preserved the endothelium-dependent vascular response to agonists following T/HS and limited syndecan-1 shedding as a marker of glycocalyx compromise (41.84 ± 9 vs. 17.63 ± 3.97 ng/mL, Shock-control vs. GM-treated, p = 0.02). Syndecan-1 cleavage was correlated with plasma trypsin-like activity (r^2^ = 0.9611). Enteral gabexate mesilate was able to maintain vascular function in experimental T/HS, which was reflected by improved hemodynamics (mean arterial pressure 50.39 ± 7.91 vs. 64.95 ± 3.43 mmHg, Shock-control vs. GM treated, p = 0.0001). Enteral serine protease inhibition may be a potential therapeutic intervention in the treatment of T/HS.

## Introduction

The ability to preserve systemic vascular function is intimately related to survival and recovery from trauma/hemorrhagic shock (T/HS). Impairment of vascular function in T/HS may lead to decreased perfusion of regional vascular beds with subsequent development of end-organ ischemia and eventual failure^1^. Thus, understanding the causes and mechanisms of vascular failure during T/HS is crucial to understanding and improving the treatment of this condition and other pathologies^2–8^.

Blood vessel walls are comprised of two major primary cell layers: the endothelium, and the principal mechanical component, vascular smooth muscle. In normally functioning arterial vasculature, the endothelium communicates with vascular smooth muscle cells (VSMCs) by continuously releasing vasoactive compounds, such as prostanoids and nitric oxide^9^. This crosstalk between components is crucial for maintaining vascular homeostasis and plays a pivotal role in low-pressure states. However, after T/HS, the response of VSMCs to vasoconstrictors is significantly attenuated, favoring relaxant stimuli^10–12^.

The luminal surface of the vascular endothelium is covered by a thin layer of gel-like material known as endothelial glycocalyx. The endothelial glycocalyx is particularly involved in the regulation of vascular permeability, microvascular tone, and coagulation^13–15^, and it is composed of glycosaminoglycans and proteoglycans, such as hyaluronan, heparan sulfate, and syndecans. Syndecans 1–4 constitute the most substantial subgroup of proteoglycans on the endothelial surface^16^. In hemorrhagic shock, this thin layer is compromised, as evidenced by increased plasma concentrations of components of the endothelial glycocalyx, specifically syndecan-1. Clinically, this is reflected in elevated resuscitation fluid requirements, edema, and increased mortality^17^.

In low-pressure states where the vasculature is highly stressed, interactions between the endothelium, VSMCs, and the endothelial glycocalyx and preservation of their function are crucial for maintaining adequate blood flow and organ viability. In the intestine, resistance mesenteric arteries (lumen diameter < 300 mm when relaxed) represent a major site of vascular resistance^18^. As such, these resistance arteries are active contributors to total systemic vascular resistance, particularly in low flow states such as circulatory shock^19,20^. Pancreatic serine proteases (e.g., trypsin and chymotrypsin) released from the ischemic intestine in the course of hemorrhagic shock are thought to be responsible for the production of factors leading to gut injury, hypotension, neutrophil sequestration in organs, and remote organ failure^21,22^. Functional assessment of the mesenteric arteries is essential for understanding the mechanisms of bowel-related ischemia in T/HS and related conditions^23^. Unfortunately, there are currently limited therapeutic options for the management and preservation of mesenteric and, thus, bowel function, as the mechanisms behind gut-related vascular failure remain largely unexplored^9^.

Our group has previously demonstrated that treatment with enteral protease inhibitors such as gabexate mesilate (GM) in experimental shock results in decreased concentrations of inflammatory mediators and protection against microvascular inflammation and early selected indicators of multiorgan failure, as well as protection against injury of the intestinal barrier and systemic hypotension^24,25^. Taken together, these findings suggest that GM may be a potential therapeutic option for the treatment of T/HS, particularly when blood products are not available. However, the mechanisms by which enteral protease inhibition is efficacious in low-pressure states are largely unexplored.

In the present study, we subjected rats to experimental trauma/hemorrhagic shock and evaluated the function of the mesenteric arteries of these animals when perfused with their own plasma. We hypothesized that enteral administration of GM preserves the vascular function of small mesenteric arteries after T/HS by decreasing circulating proteolytic activity^26^. Improvements in blood pressure after enteral protease inhibition are associated with enhanced autonomic function and a preserved vasoconstrictor response^25^.

## Results

### Effect of enteral serine protease inhibition on systemic hemodynamics

Animals treated with enteral GM were able to increase and maintain blood pressure with crystalloid-only resuscitation after T/HS compared to the Shock-control animals. Systolic arterial pressure, in particular, was greater in the GM-treated group by almost 20 mmHg after the resuscitation period (p = 0.0004). Diastolic arterial pressure was also greater in the GM-treated compared to the Shock-control group after fluid resuscitation (p = 0.0068), resulting in an improved mean arterial pressure (MAP) in the GM-treated group (Table 1). This improvement in blood pressure was observed despite a decreased fluid infusion requirement in GM-treated animals. Although rats from both groups had similar body weights (~ 408 g) and had approximately 55% of their estimated blood volume removed to induce hemorrhagic shock (~ 13 mL), the shock-control group required 1.93 times the volume of lactate ringer compared to 1.12 times in the GM-treated group to achieve hemodynamic stability. No differences were found in heart rate or arterial blood gas analyses between groups. The complete set of hemodynamic and arterial blood gas analyses are provided as supplementary material.

**Table 1:** Experimental parameters.

Variable	Shock-control	GM-treated	p value
Body weight (g)	408.88 ± 33.90	395.25 ± 19.23	0.3224
Blood removed (mL)	13.08 ± 1.75	12.50 ± 1.20	0.4310
Lactated Ringer infused (mL)	25.11 ± 4.62	14.00 ± 1.85****	< 0.0001
Volume infused (%)	193.74 ± 33.83	112.91 ± 17.44****	< 0.0001
Systolic arterial pressure (mmHg)	81.69 ± 11.54	101.25 ± 5.84***	0.0004
Diastolic arterial pressure (mmHg)	32.18 ± 9.42	43.19 ± 4.14**	0.0068
Mean arterial pressure (mmHg)	50.39 ± 7.91	64.95 ± 3.43***	0.0001
Pulse pressure (mmHg)	49.05 ± 15.64	58.06 ± 7.78	0.1539
Heart rate (bpm)	378.46 ± 31.24	394.49 ± 33.77	0.3005
pH	7.42 ± 0.15	7.45 ± 0.04	0.5254
paCO_2_ (mmHg)	36.24 ± 12.04	34.53 ± 2.72	0.7002
paO_2_ (mmHg)	104.32 ± 23.72	100.20 ± 8.39	0.6462
HCO_3_ (mmol/L)	22.63 ± 4.89	24.93 ± 5.14	0.3355
BE (B)	− 1.74 ± 6.19	0.40 ± 3.26	0.3871
O_2_ saturation (%)	94.28 ± 8.49	94.77 ± 1.27	0.8742
Hemoglobin (g/dL)	5.99 ± 0.90	7.02 ± 1.03*	0.0331
Blood lactate (mmol/L)	8.72 ± 4.17	7.80 ± 2.60	0.5902
Plasma Lactate (mmol/L)	7.93 ± 1.17	7.23 ± 0.93	0.7467

In-vivo biological parameters between experimental trauma/hemorrhagic shock animals reperfused with Lactated Ringer’s solution in the presence (GM-treated) or in the absence (Shock-control) of enteral gabexate mesilate. Potential of hydrogen (pH), partial pressure of carbon dioxide in arterial blood (PaCO_2_), partial pressure of oxygen in the arterial blood (PaO_2_); bicarbonate (HCO_3_); base excess (BE). p-values of GM-treated vs. Shock-control group obtained by unpaired *t* test. *p < 0.05, **p < 0.01, ***p < 0.001, ****p < 0.0001.

### Effect of enteral serine protease inhibition on vascular contractility

**Figure 1:**
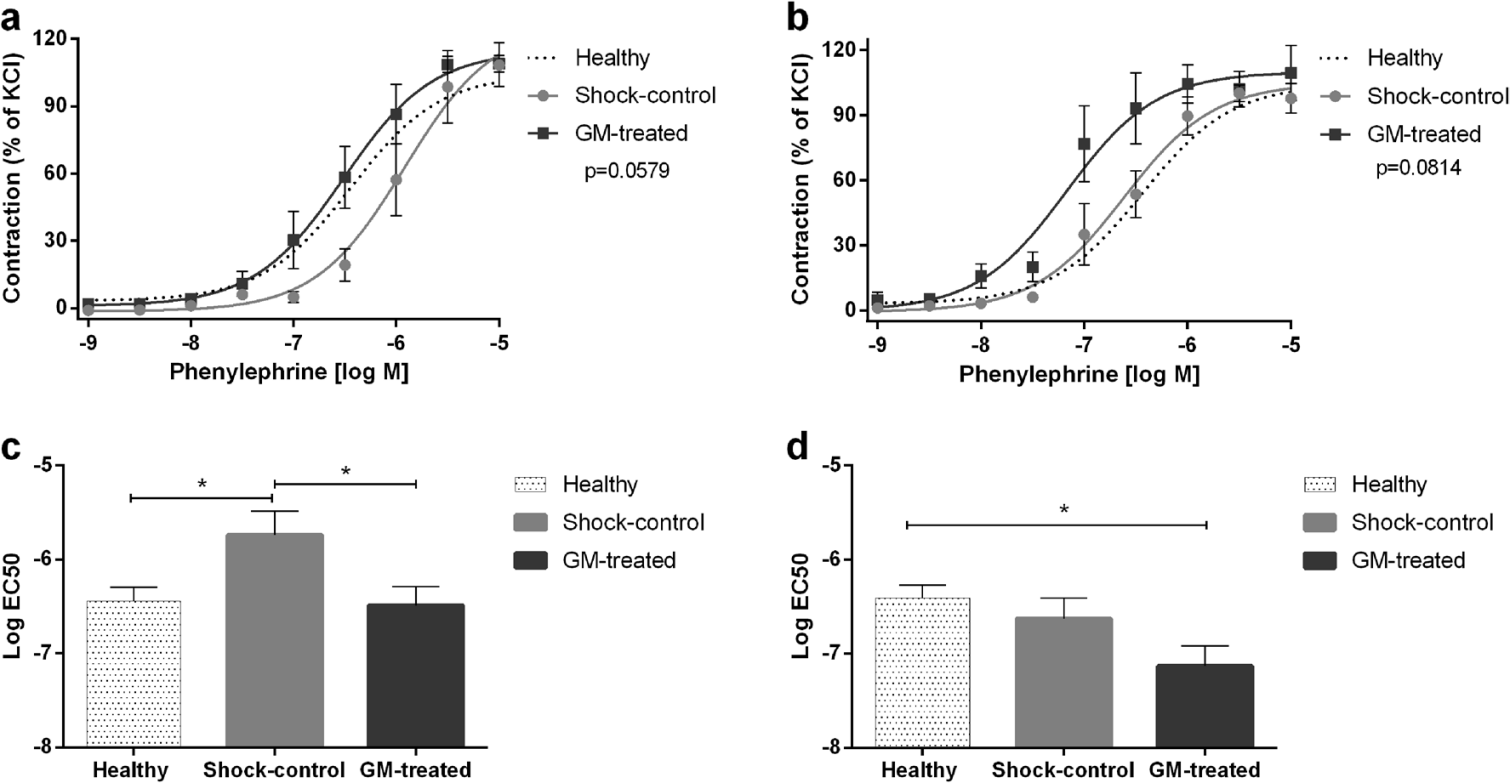
Vasoconstriction response. Ex-vivo mesentery artery response to phenylephrine. (**a**) Concentration–response curve to phenylephrine in shock arteries perfused with plasma from shocked animals; (**b**) Concentration–response curve to phenylephrine in healthy arteries perfused with plasma from shocked animals (previously reported^25^); (**c**) Half maximal effective concentration (logEC_50_) of phenylephrine on shock arteries perfused with plasma from shocked animals; (**d**) logEC_50_ of phenylephrine on healthy arteries perfused with plasma from shocked animals. p-value on (**a**) and (**b**) represents the curve behavior by two-way ANOVA for repeated measurements, GM-treated vs. Shock-control group. The traced line shows a healthy vessel dose–response pattern, not included in the statistical analyses. *p < 0.05 by one-way ANOVA.

Arteries from non-treated (Shock-control group) rats after T/HS perfused with autologous plasma showed an impaired concentration–response curve (CRC) to phenylephrine (PE)-induced vasoconstriction when compared to arteries from healthy animals perfused with autologous plasma. In contrast, arteries from GM-treated animals perfused with autologous plasma displayed similar behavior to those of healthy animals after PE stimulation, demonstrating a protective role for enteral serine protease inhibition in the preservation of contractile function (Fig. 1a). No impairment in contractility was observed in healthy arteries when perfused with plasma from Shock-control rats. However, when healthy arteries were perfused with plasma from GM-treated shock animals, contractility induced by PE was enhanced compared to the Shock-control group or healthy plasma (Fig. 1b). This finding was previously reported^25^ and is displayed here for completeness. Drug sensitivity (represented by − logEC_50_) reflected the behavior described above: non-treated (Shock-control) shock arteries perfused with autologous plasma were less sensitive to PE compared to the GM-treated group (− logEC_50_ − 5.73 ± 0.25 vs. − 6.48 ± 0.2, Shock-control vs. GM-treated, p = 0.04, Fig. 1c), and healthy arteries demonstrated increased sensitivity to PE when perfused with plasma from shocked rats treated with the protease inhibitor (− logEC_50_ − 6.62 ± 0.21 vs. − 7.13 ± 0.21, Shock-control vs. GM-treated, p = 0.02, Fig. 1d).

### Effect of enteral serine protease inhibitor on endothelium-dependent vascular dilation

**Figure 2:**
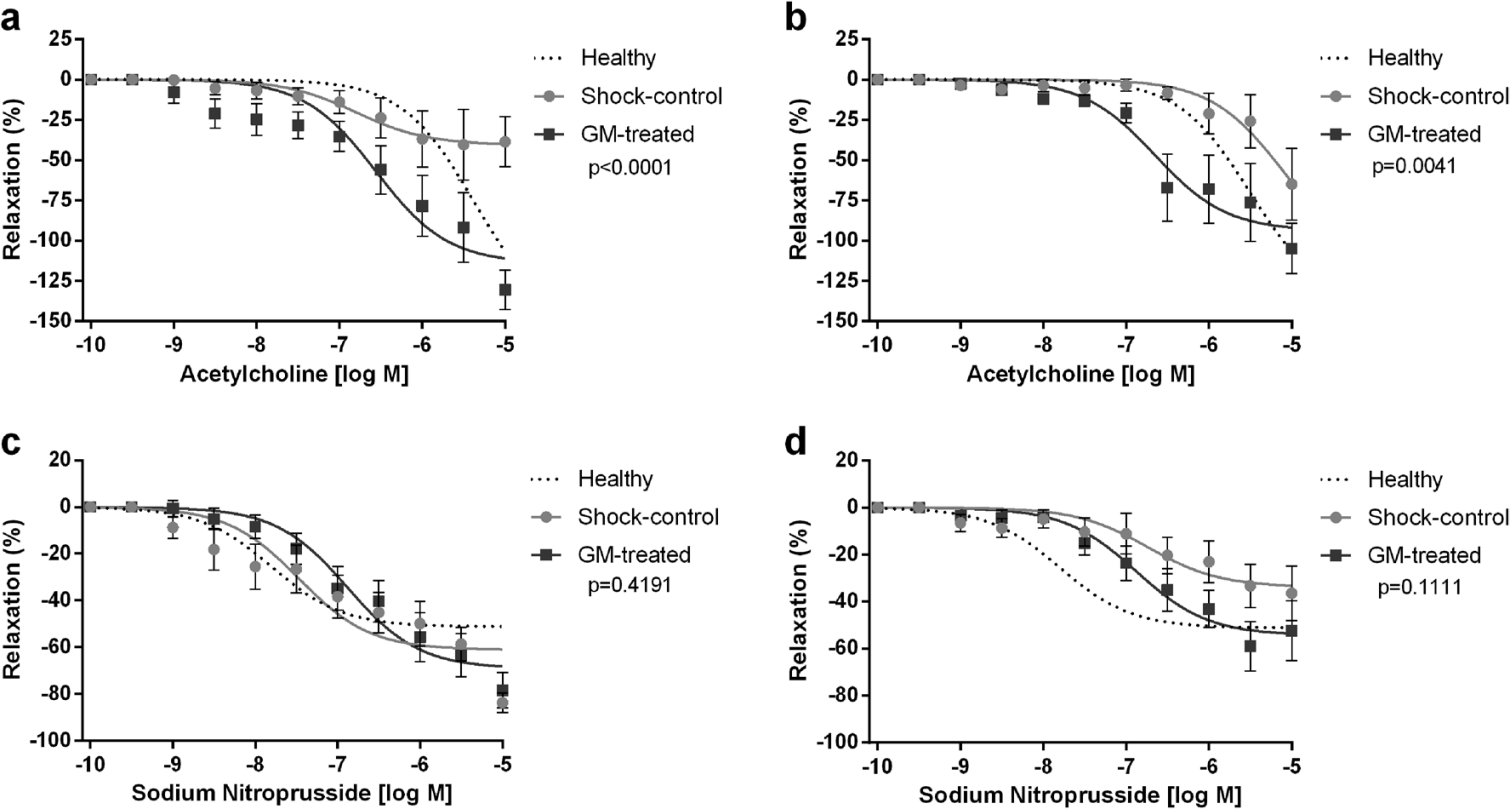
Vasodilatory response. Ex-vivo mesentery artery response to acetylcholine (ACh) and sodium nitroprusside (SNP). (**a**) Concentration–response curve to acetylcholine in shock arteries perfused with plasma from shocked animals; (**b**) Concentration–response curve to acetylcholine in healthy arteries perfused with plasma from shocked animals; (**c**) Concentration–response curve to sodium nitroprusside in shock arteries perfused with plasma from shocked animals; (**d**) Concentration–response curve to sodium nitroprusside in healthy arteries perfused with plasma from shocked animals (previously reported^25^). The p-value represents the curve behavior by two-way ANOVA for repeated measurements, GM-treated vs. Shock-control group. The traced line shows a healthy vessel dose–response pattern, not included in the statistical analyses.

CRCs to acetylcholine (ACh) were constructed to evaluate the endothelium-dependent response to exogenous stimulation. Enteral serine protease inhibition preserved endothelial-dependent vascular function following T/HS in mesenteric arteries, as shown in Fig. 2a. In contrast, an impaired endothelium-dependent response was observed in the Shock-control group (p < 0.0001 between groups). Interestingly, healthy arteries, when perfused with plasma from Shock-control animals, exhibited an impaired CRC to ACh, i.e., plasma from rats subjected to experimental T/HS induced endothelial dysfunction in healthy arteries, which was again prevented by enteral treatment with the serine protease inhibitor (p = 0.0041 between groups, Fig. 2b).

### Effect of enteral serine protease inhibition on endothelium-independent vascular dilation

A concentration–response curve to sodium nitroprusside (SNP) was performed to evaluate endothelium-independent response in mesenteric arteries. Arteries from both groups (Shock-control and GM-treated) and healthy arteries perfused with shock plasma (treated or not, previously reported^25^ and displayed here for completeness) demonstrated an intact response to SNP, displaying similar behavior to the healthy control group (Fig. 2c,d). Thus, experimental T/HS did not alter the endothelium-independent response, regardless of the treatment group.

### Effect of enteral serine protease inhibition on circulating (systemic) proteases

**Figure 3:**
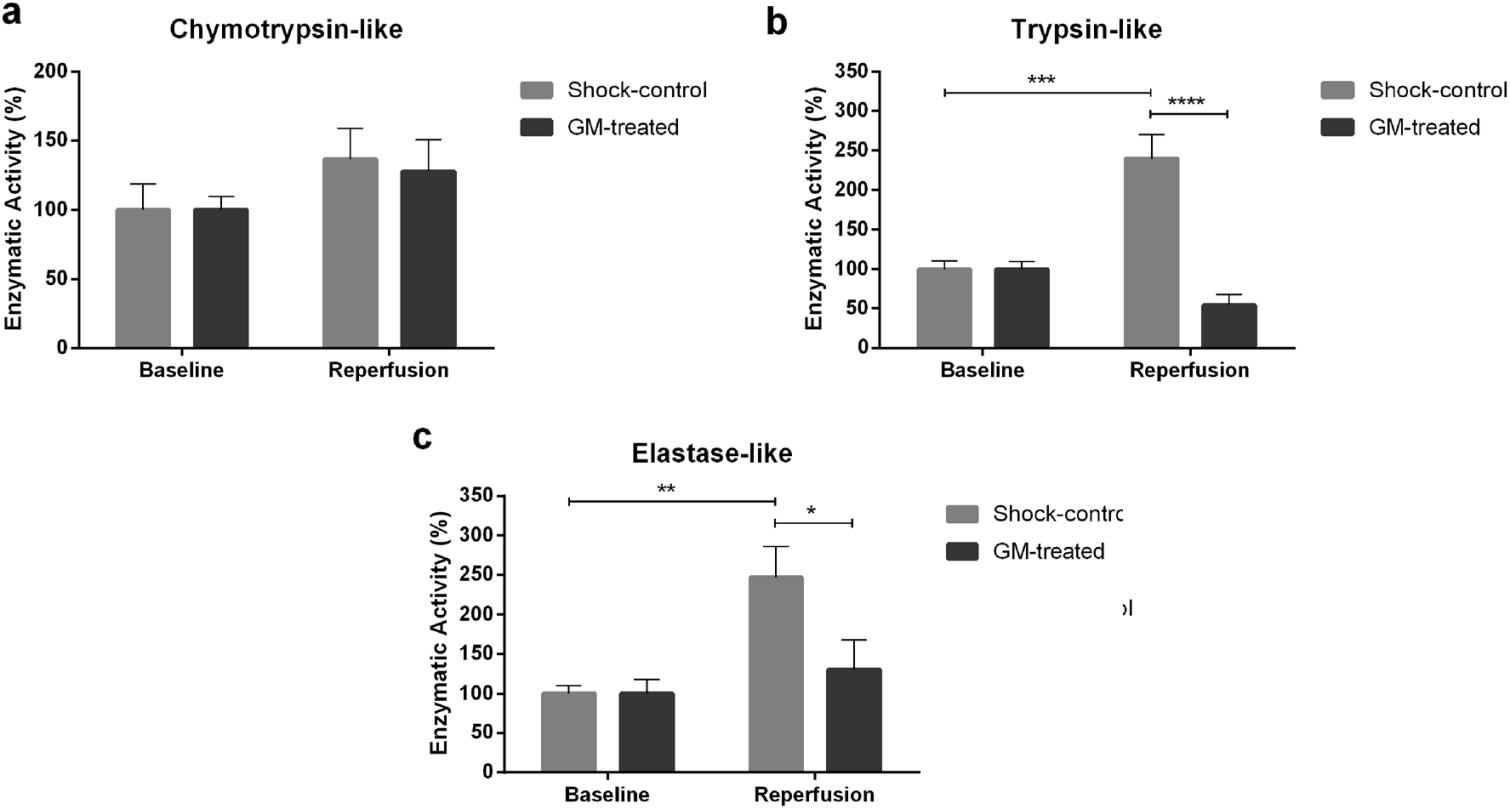
Enzymatic activity. Plasma enzymatic activity was measured by the fluorescent substrate for (**a**) Chymotrypsin-like activity; (**b**) Trypsin-like activity; (**c**) Elastase-like activity. Values normalized by baseline levels of activity. *p < 0.05, **p < 0.01, ***p < 0.001, ****p < 0.00001 by two-way ANOVA for repeated measurements.

**Figure 4:**
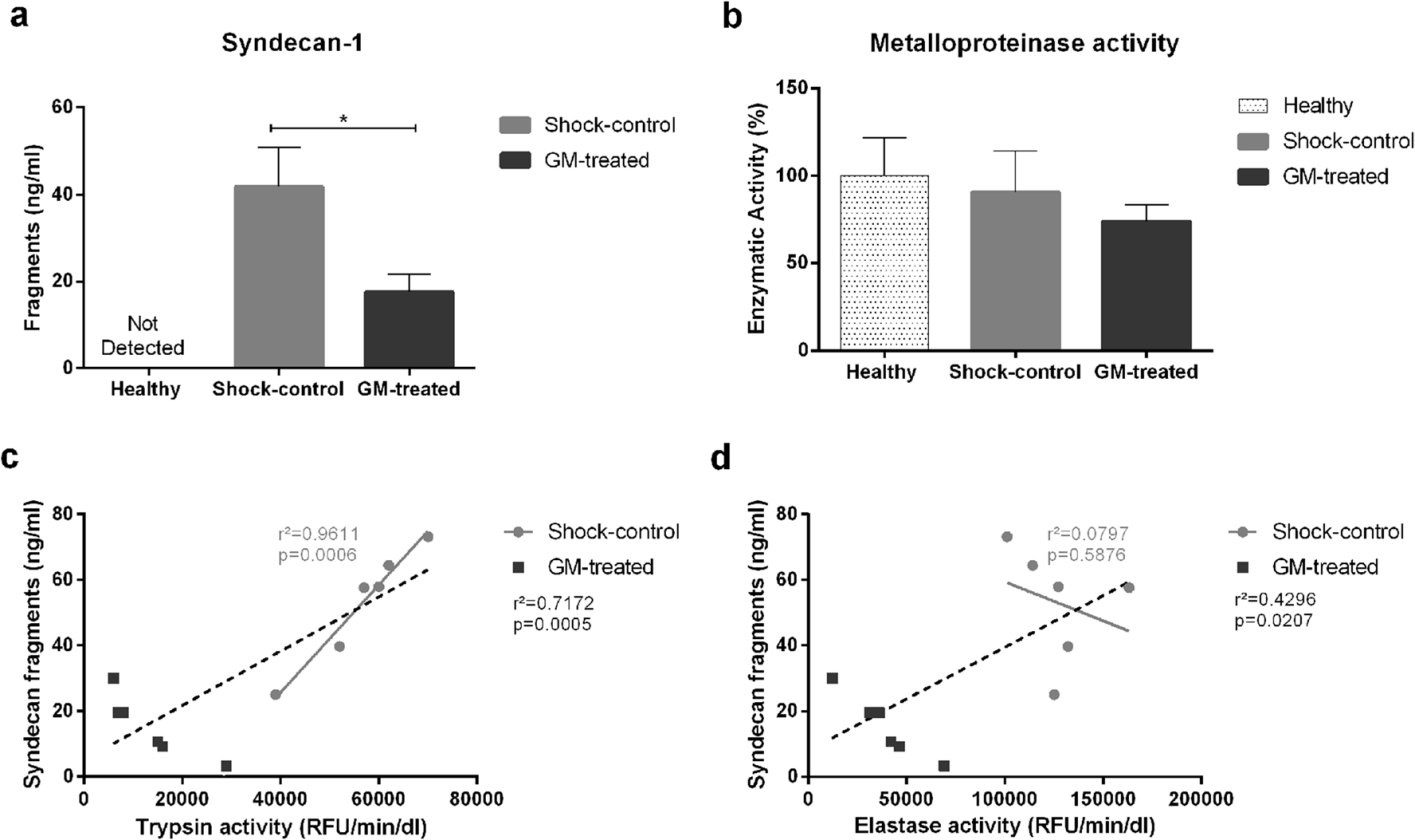
Glycocalyx shedding. Interaction of enzymatic activity and glycocalyx shedding after trauma/hemorrhagic shock. (**a**) Glycocalyx damage measured by syndecan-1 fragments on plasma; (**b**) Total matrix metalloproteinase (MMP) activity on plasma, normalized by activity on healthy animals; (**c**) Correlation analyses of trypsin-like activity and syndecan fragments; (**d**) Correlation analyses of elastase-like activity and syndecan fragments. *p < 0.05 GM-treated vs. Shock-control group obtained by unpaired *t* test. r^2^ and p-values refer to the determination factor and significance value of the entire analysis. The light-grey values represent the Shock-control group only.

After experimental T/HS followed by reperfusion, levels of trypsin-like and elastase-like activity were increased 2.5-fold in the plasma of the Shock-control group (trypsin-like activity Shock-control 100 ± 10.18 vs. 240.03 ± 30.25, baseline vs. reperfusion, p = 0.0001; elastase-like activity Shock-control 100 ± 9.98 vs. 246.56 ± 39.09, baseline vs. reperfusion, p = 0.009). Enteral treatment with GM was able to reduce these activity levels to baseline (trypsin-like activity after reperfusion: 240.03 ± 30.25 vs. 53.89 ± 13.79, Shock-control vs. GM-treated, p < 0.0001; elastase-like activity after reperfusion: 246.56 ± 39.09 vs. 130 ± 37.56, Shock-control vs. GM-treated, p = 0.04, Fig. 3b,c). No significant changes were observed in chymotrypsin-like activity (Fig. 3a) or total matrix metalloproteinase (MMP) activity (Fig. 4b) in this T/HS model.

### Effect of enteral serine protease inhibition on the endothelial glycocalyx

Plasma syndecan-1 was significantly elevated in Shock-control animals compared to GM-treated rats (41.84 ± 9 vs. 17.63 ± 3.97 ng/mL, p = 0.04) and was undetectable in healthy animals (Fig. 4a). Correlation analyses demonstrate an association of syndecan-1 cleavage with trypsin-like activity (r^2^ = 0.9611) and no correlation with elastase-like activity (r^2^ = 0.0797, Fig. 4c,d).

## Discussion

This study demonstrates that the enteral administration of gabexate mesilate (GM) in T/HS was able to (a) preserve endothelial function in ex-vivo perfused mesenteric arteries, (b) limit endothelial glycocalyx shedding, and (c) normalize the vasoconstrictor response to PE, in addition to its previously described effects on blood pressure improvement^25^.

The physiological role of the endothelium in critical illness has been widely investigated, and it is acknowledged to play a vital role in preserving vascular homeostasis^27^. However, during T/HS, many factors, including reduced oxygen availability and elaboration of inflammatory mediators, lead to endothelial cell dysregulation and subsequent impairments in vascular function, including endothelial glycocalyx shedding, the latter of which results in increased vascular permeability and leakage of fluids into tissues^28^. The microcirculation—particularly in the gastrointestinal tract—plays a pivotal role in T/HS. As a consequence of microvascular ischemia, intestinal barrier function can be severely altered, triggering a systemic inflammatory response that may result in multi-organ failure^29^. A proposed mechanism for systemic vascular and organ failure is the leakage of intestinal proteases into the circulation, causing systemic proteolysis^30^ and producing vasoactive mediators that may lead to or exacerbate the shock condition^31^. In this context, enteral treatment with GM was able prevent injury by blocking protease activity in the small bowel and maintaining improved bowel homeostasis.

To assess the microcirculatory endothelium-dependent vascular response, we performed acetylcholine (ACh) concentration–response curves in mesenteric resistance arteries from rats. Shocked animals that received enteral GM treatment demonstrated preserved endothelial function, as measured by complete relaxation of the vessel in response to the ACh challenge, while the Shock-control group showed an impaired ACh response (Fig. 2a). Interestingly, arteries from healthy animals perfused with plasma from shocked animals displayed a diminished endothelium-dependent response. In contrast, arteries from healthy animals perfused with plasma from protease inhibitor-treated shocked animals demonstrated preserved endothelial function (Fig. 2b). These observations point to the role of circulating mediators present in plasma in shock and suggest that humoral factors may cause the endothelial dysfunction seen in T/HS.

Using a pool of fluorescently labeled peptide substrates, we were able to identify the overall serine protease activity in plasma. Trypsin-like and elastase-like activity increased by around 150% in the Shock-control group compared to their baseline levels (Fig. 3b,c). Further, enteral GM treatment significantly reduced the enzymatic activity levels to near basal values. This is suggestive of the possible intestinal origin of the observed systemic enzymatic activity, as previously proposed by our group^31^, and supportive of the efficacy of enteral inhibition as a treatment to reduce dysregulated enzymatic activity and the subsequent damage to protein systems and tissues^26^.

A high concentration of syndecan-1 was observed in the plasma after T/HS but was undetectable in the circulation of healthy animals. GM treatment reduced syndecan-1 levels by approximately 50% compared to the Shock-control group (Fig. 4a). Glycocalyx degradation is usually associated with matrix metalloproteinases (MMP) activity^32–36^. From our results, it is unclear as to whether circulating MMPs play a significant role in this process, as the total plasma MMP activity was comparable between all three groups (Fig. 4b).

Using correlation analyses, we could establish that syndecan cleavage is highly associated with trypsin-like activity. In Fig. 4c,d, animals from the GM-treated and the Shock-control groups occupy very distinct positions on the dispersion graphs. Higher enzymatic activity is directly correlated with syndecan-1 plasma levels. Although both trypsin and elastase activities are related to syndecan-1, trypsin-like activity shows a much stronger correlation (r^2^ = 0.9611) in the Shock-control group. Based on these data, we suggest that T/HS systemically increases enterally derived serine protease activity. Trypsin-like proteases may then be able to directly cleave the glycocalyx without the involvement of MMPs, leading to endothelial dysfunction. Here we demonstrate that enteral treatment with GM preserves endothelial function and reduces glycocalyx shedding.

Endothelial glycocalyx shedding has been shown to occur in response to endotoxin^37^, serine proteases^38–40^, and complement activation^41^; targeting the enzymes responsible for glycocalyx shedding may act to preserve endothelial function. Our results are consistent with previous studies in rodents demonstrating preservation of the endothelium via metalloproteinase inhibition in the gut^42^ and in patients with traumatic intracranial hemorrhage who received early treatment with a protease inhibitor resulting in decreased levels of circulating syndecan-1^43^.

One of the functions of the endothelial glycocalyx is to serve as a receptor for both chemical and physical signals, triggering the physiological responses of the vascular endothelium. Glycocalyx components, particularly syndecan-1 are known to act as mechanosensors and mechanotransmitters regulating flow-induced nitric oxide release^16,44,45^. Destruction of the glycocalyx leads to capillary leakage, edema, accelerated inflammation, platelet aggregation, hypercoagulation and a decrease in vascular responsiveness^46^. Considering the role of the glycocalyx in vascular function, its preservation during T/HS may have a positive impact on inflammation and endothelial physiological responses, leading to better outcomes. Although it had been previously proposed that T/HS resuscitation must contain blood to preserve endothelial function^47^, this study demonstrates that enteral treatment with the protease inhibitor gabexate mesilate is able to largely conserve endothelial function in the absence of blood product resuscitation.

Diminished responsiveness of vascular smooth muscle to vasopressors administered to restore and maintain blood pressure is often observed following severe T/HS^12^. We have previously shown that this responsiveness may be due—at least in part—to activation of the Toll-like receptor 4 (TLR4), which in turn reduces the levels of membrane α-1 adrenergic receptor^48^, a primary mediator of vascular smooth muscle contraction^49^. This decreased α_1_ adrenergic receptor responsiveness and density can be mitigated by enteral protease inhibition^25,50^ along with a reduction in inflammatory mediator concentrations^51^. In the current study, rats receiving enteral treatment with the protease inhibitor had their concentration–response curve to PE (α-1 receptor agonist) shifted to the left, indicating a normalized response to this vasopressor after T/HS (Fig. 1a).

Endothelial glycocalyx shedding can also activate inflammation via activation of TLR4; fragments from the glycocalyx stimulate the release of proinflammatory cytokines and increase inflammation through TLR4^52^. In the T/HS context, TLR4 may be activated as a consequence of the glycocalyx shedding which in turn reduces the levels of membrane α-1 adrenergic receptor, influencing the vasoconstriction response.

The precise mechanism by which vasoconstriction is enhanced in the GM-treated group, whether via direct action on α-1 receptors in vascular smooth muscle or via protection of the endothelial glycocalyx, remains unexplored, but it is possible that enteral protease inhibition mitigates the leakage of gut proteases into the circulation and that proteases may play a role, either directly or indirectly, by increasing inflammation and inflammatory mediators that bind to receptors such as TLR4, resulting in decreased α-1 concentrations and diminished vasoconstriction.

Another hypothesis to explain the enhanced response to PE in GM-treated animals is the possible synergism of higher catecholamine concentrations observed in plasma of GM-treated animals necessitating reduced exogenous pressor requirements^25^. In support of these assumptions is the fact that healthy arteries displayed an even greater vasoconstriction when perfused with plasma from GM-treated shock animals, and moreover showed a higher sensitivity to PE when compared with both Shock-control and sham control groups (Fig. 1b,d). Regarding smooth muscle vasorelaxation, in agreement with other studies, we show that endothelial-independent relaxation remains functional after hemorrhagic shock^1,47,53^ regardless of treatment (Fig. 2c,d).

Taken together, these results demonstrate that enteral serine protease inhibition in T/HS results in a reduction of circulating proteases, decreased need for aggressive fluid during resuscitation, improved response to vasopressors, limited glycocalyx damage/shedding, and preserved endothelial function, leading to improved hemodynamics following shock.

## Conclusion

Enteral administration of the protease inhibitor gabexate mesilate promotes vascular protection of resistance mesenteric arteries during T/HS, which are integral to the maintenance of systemic blood pressure. Enteral serine protease inhibition may be an effective modality for the prevention of vascular failure, leading to improved outcomes after circulatory shock.

## Materials and methods

### Experimental protocol

Wistar male rats (380–440 g, Charles River Laboratories, San Diego, CA, USA) were randomly assigned to a Shock-control group (n = 6) or GM-treated group (n = 6). The Shock-control group received enteral vehicle alone (Golytely^®^), while the treated group received enteral infusion of the serine protease inhibitor gabexate mesilate (GM) in the vehicle. An isolated chamber with 5% isoflurane was used to induce anesthesia, followed by nose cone anesthetic delivery at a maintenance level of 1.5% isoflurane in 21% oxygen concentration (FiO_2_) at a flow rate of 0.8 L/min.

### Trauma/hemorrhagic shock protocol

For the T/HS experiments, blood withdrawal and intravenous (IV) fluid therapy (PE 50 tubing) were conducted via right femoral vein cannulation, and blood pressure and heart rate monitored (PE 10 tubing) via right femoral artery cannulation. Heart rate and blood pressure were continuously recorded using the Power Lab^®^ data acquisition system on LabChart 7.0 software with a sampling frequency of 2 kHz/channel (ADInstruments, Dunedin, New Zealand) as previously described^25^. To ensure stable respiratory function, partial pressure of arterial oxygen content (PaO_2_) and carbon dioxide (PaCO_2_) were measured regularly. A water-heated platform was used to maintain optimal body temperature (37 °C) and temperature was monitored throughout the experiment by a rectal probe.

Trauma was characterized by laparotomy. Enteral infusion of GM and/or vehicle was facilitated by oral insertion of a double-lumen enteral catheter consisting of an inflow tube for solution infusion (PE 50 tubing with a 3 cm Tygon^®^ guide tip, inner diameter of 0.8 mm) and an outflow tube (PE 10 tubing with orifice located in the distal esophagus) to prevent reflux, with both tubes connected to an external peristaltic pump, adapted from Aletti et al.^54^ Enteral infusion of the carrier solution Golytely^®^ (0.14 g/mL sterile water, 110 μL/min for 150 min) was started 20 min into the hypovolemic period for all animals, in order to optimize enteral flow rates necessary to fill the small bowel during the shock and reperfusion periods without appreciable aspiration risk^54^. Gabexate mesilate (10 mg/kg) was added to the enteral carrier solution in the GM-treated group but not in the Shock-control group. The GM dose of 10 mg/kg was established based on pilot experiments from our group, using published intravenous dosing as a baseline. The main investigator was blinded to treatment assignment.

Heparin (100 units/kg) was given to all animals intravenously to prevent clotting of catheters, and after abdominal cavity suture, the animals were observed for 20 min to define baseline hemodynamics. Hemorrhage was induced by removing blood from the femoral vein (0.5 mL/min) until the MAP reached 40 mmHg. Removal or return of small aliquots of blood was done as necessary to maintain MAP between 35–40 mmHg for 90 min, at which time animals were resuscitated with IV infusion of Lactated Ringer’s (LR) solution containing 5% dextrose, administered 2 mL/min to a target MAP ≥ 60 mmHg. Animals were monitored for an additional 120 min, receiving fluid as necessary to maintain MAP, before being euthanized (B-Euthanasia, 120 mg/kg), which was confirmed by a complete loss of blood pressure signal and adjunctive bilateral thoracotomy. Presented below is a flowchart depicting the T/HS protocol.
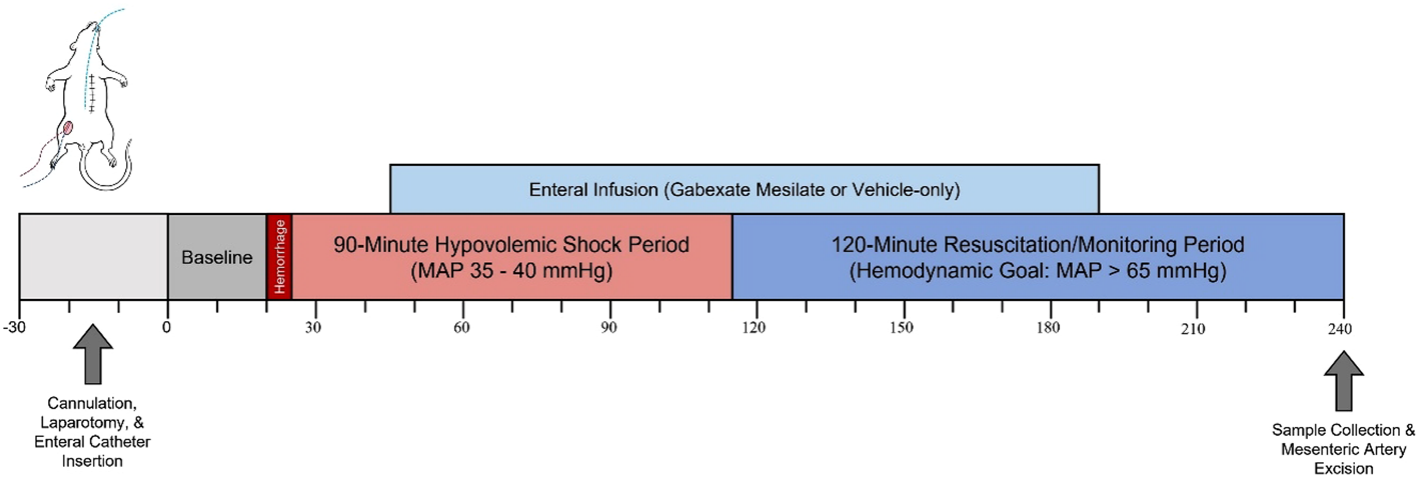


### Ex-vivo vascular reactivity

Vascular reactivity was accessed by evaluation of ex vivo mesenteric artery function using a pressure myograph. 3–5 mm arterial third-order segments from mesenteric arteries (150–300 µm diameter) were cannulated with glass micropipettes in a servo-controlled pressurized myograph chamber (Living Systems Instrumentation, St Albans, VT) containing 6 mL of Krebs solution at 37 °C, pH = 7.4 and maintained under constant aeration (95% O_2_:5% CO_2_). Intraluminal pressure was slowly increased to 140 mmHg using Krebs solution, while the artery was gradually stretched until it reached optimal diameter and tension, at which point the pressure was subsequently decreased to 70 mmHg for a 1-h stabilization period where it remained unchanged for the duration of the experiment^55^.

Following the stabilization period, arterial segments from GM-treated or Shock-control rats were perfused with their own (autologous) plasma obtained prior to euthanasia and allowed to stabilize for 30 min (n = 6 all groups). The arteries were then challenged with phenylephrine (PE), sodium nitroprusside (SNP) and acetylcholine (ACh). To determine the contribution of circulating plasma factors versus non-circulating tissue factors in vascular function, a second set of experiments was conducted. Arteries from healthy non-shocked rats were perfused with plasma obtained from shocked rats, either treated with GM or not (Shock-control), and then subjected to the same challenges are as the first set of experiments (n = 6 all groups). Results from PE and SNP experiments examining the responses of healthy arteries were previously reported and are included in the analysis for completeness^25^. In all cases, a control group consisting of healthy arteries perfused with autologous plasma was included.

The experiments were conducted as previously described^25^. In brief, after the stabilization period with plasma, potassium chloride 75 mM (KCl) was added to the bath chamber and only vessels displaying vasoconstriction of 50% or more were used. Concentration response curves (CRCs) were constructed by the cumulative addition of PE, SNP or ACh at concentrations ranging from 10^−9^ to 10^−5^ M in half-log increments to the mounted arteries. After each set of measurements, the bath chamber was washed with fresh Krebs solution and left to stabilize for 20 min. Arterial contraction and dilation measurements are expressed as a percentage of the maximum contraction to KCl. The arteries were viewed with a 10 × objective equipped with a monochrome video charge-coupled device camera, and their luminal diameter recorded continuously by image capture with a video frame grabber and real-time edge-detection system (VasoTracker 1.0.3)^56^. Below is a representation of the groups for both sets of experiments.
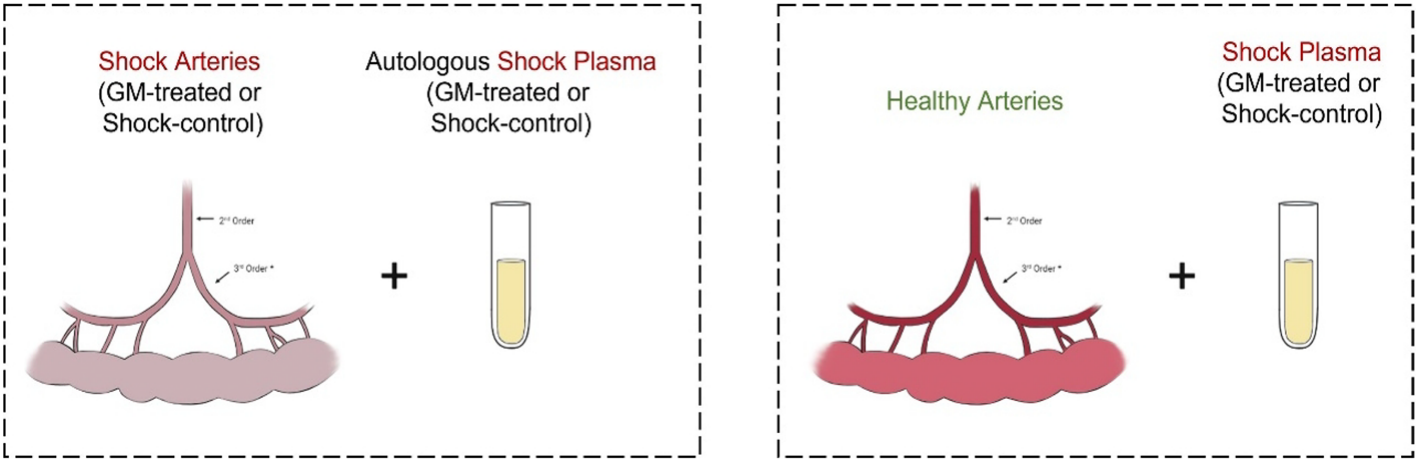


### Plasma syndecan-1

Syndecan-1 in rat plasma samples was measured by ELISA (MBS2703971, MyBioSource). Plasma samples were diluted in PBS 1:5 and were repeated in triplicate in accordance with the manufacturer's instructions.

### Plasma enzymatic activity assay

After diluting plasma samples in PBS (1:50), 10 µL of sample was added to 10 µL of fluorescent substrate [10 µM] and fluorescence kinetics were immediately measured using a spectrophotometer (FilterMax F5 Multi-Mode, Molecular Devices, San Jose, CA) and enzymatic activity was measured [relative fluorescence units per second per microliter of plasma (RFU/s/µL)]. Results are presented as percentage of baseline activity. A library of proteolytically-cleaved substrates was used [2–5 amino acid sequence, tagged with the fluorescent marker 7-Amino-4-methylcoumarin (AMC)]. We considered activity to be trypsin-like for cleavages occurring at arginine/lysine, chymotrypsin-like for tryptophan/phenylalanine/tyrosine, and elastase-like for alanine/valine/serine/glycine/leucine/isoleucine. A complete list of substrates and sequences used can be found in Supplementary Material.

### Matrix metalloproteinase activity assay

MMP activity was measured using a non-specific MMP fluorescence resonance energy transfer peptide substrate (ab112147, Abcam) according to the manufacturer’s instructions. Plasma samples were repeated in triplicate with a final dilution of 1:10. The use of recombinant MMP2 and MMP9 as positive controls and a broad spectrum MMP inhibitor (Sigma-Aldrich GM6001) as a negative control confirmed the viability of the assay.

### Statistical analysis

Blinded to the investigators, animals were randomly allocated to experimental groups and treatment (GM-treated vs Shock-control). Data were analyzed using GraphPad Prism 7^®^ (GraphPad Software, Inc., San Diego, CA). After testing via the Shapiro–Wilk normality test, the data were subjected to one-way or two-way Analysis of Variance (ANOVA) for repeated measurements. When appropriate, post hoc analyses were performed using the Bonferroni multiple comparisons test. A linear regression with correlation between enzymatic activity and proteolysis was also conducted. Results are presented as mean ± standard error and p < 0.05 were considered significant.

### Ethical statement

All experiments were carried in accordance with University of California, San Diego Institutional Animal Care and Use Committee approvals (protocol number S16062 from 05/17/2017) and conform to the Guide for the Care and Use of Laboratory Animals, 8th edition, by the United States National Institutes of Health (2011). In addition, this study has been conducted and reported in compliance with the ARRIVE (Animal Research: Reporting of In Vivo Experiments) guidelines, ensuring transparency and reproducibility in reporting the experimental design, animal welfare, and statistical analysis.

## Supplementary Information

Supplementary Figures.

## Data Availability

The datasets generated and/or analyzed during the current study are available in a public OneDrive folder repository, https://1drv.ms/f/s!AqT_EaB6sAlFg95T8sztSlsVTsoKJg?e=YccGWp.
