# Supplementary material for: Enteral administration of the protease inhibitor gabexate mesilate preserves vascular function in experimental trauma/hemorrhagic shock
Source: Sci Rep. 2023 Jun 22;13:10148. doi: 10.1038/s41598-023-36021-7 (PMC10287748; doi:10.1038/s41598-023-36021-7)
Supplement: Supplementary file 1 — Supplementary Figures. [file 41598_2023_36021_MOESM1_ESM.docx]

***Enteral administration of the protease inhibitor gabexate mesilate preserves vascular function in experimental trauma/hemorrhagic shock.***

*Nathalia, J.D. Moreira, M.A.^1^, Fernando dos Santos, Ph.D.^2^, Joyce B. Li, B.S.^3^, Federico Aletti, Ph.D.^4^, Maria Claudia C. Irigoyen M.D., Ph.D.^1^, Erik B. Kistler, M.D., Ph.D.^2,5^

^1^Instituto do Coração, Hospital das Clínicas, Faculdade de Medicina, Universidade de São Paulo, São Paulo, Brazil

^2^Department of Anesthesiology & Critical Care, University of California, San Diego, La Jolla, CA, USA

^3^Department of Bioengineering, University of California, San Diego, La Jolla, CA, USA

^4^Universidade Federal de São Paulo, São José dos Campos, Brazil

^5^Veterans Affairs San Diego Healthcare System, San Diego, CA, USA

Emails: [nathaliajuocys@hotmail.com](mailto:nathaliajuocys@hotmail.com), [fedossantos@health.ucsd.edu](mailto:fedossantos@health.ucsd.edu), [jbli@eng.ucsd.edu](mailto:jbli@eng.ucsd.edu), [faletti@unifesp.br](mailto:faletti@unifesp.br), [hipirigoyen@gmail.com](mailto:hipirigoyen@gmail.com), [ekistler@ucsd.edu](mailto:ekistler@ucsd.edu)

**Supplementary graphs**

Supplementary file with the complete set of hemodynamic (**Figure 1**) and arterial blood gas analyses (**Figure 2**). Data collected from the beginning of the experiment, before blood removal to induce shock (Baseline), and at the end of the in vivo procedure (Final), 120 minutes after reperfusion. Results are shown by mean ± standard error. Statistical analyses was done by Two-way ANOVA.

**Figure 1: Hemodynamics.** *p<0.05, **p<0.01, ***p<0.001, ****p<0.0001.

**Figure 2: Arterial blood gas**. **A** Potential of hydrogen (pH), **B** partial pressure of carbon dioxide in arterial blood (PaCO_2_), **C** partial pressure of oxygen in the arterial blood (PaO_2_), **D** bicarbonate (HCO_3_), **E** base excess (BE), **F** oxygen saturation (O_2_ saturation), **G** Hemoglobin levels, **H** Lactate. *p<0.05, **p<0.01, ***p<0.001, ****p<0.0001.
